# Systemic expression of Alu RNA in patients with geographic atrophy secondary to age-related macular degeneration

**DOI:** 10.1371/journal.pone.0220887

**Published:** 2019-08-19

**Authors:** Hiroyuki Yoshida, Tokiyoshi Matsushita, Erika Kimura, Yukie Fujita, Robert Keany, Toshihiro Ikeda, Masanao Toshimori, Takahiro Imanaka, Masatsugu Nakamura

**Affiliations:** 1 Research and Development Division, Santen Pharmaceutical Co., Ltd., Osaka, Japan; 2 Research and Development Division, Santen Inc., Emeryville, CA, United States of America; University of Florida, UNITED STATES

## Abstract

Geographic atrophy (GA) secondary to age-related macular degeneration (AMD) is characterized by irreversible loss of macular retinal tissue and retinal pigment epithelium (RPE) cells. Several studies have revealed that accumulation of Alu RNA in RPE cell causes RPE cell degeneration in AMD. In the present study, systemic Alu RNA expression levels were determined in 33 subjects with GA and 40 control subjects using a proprietary Alu RNA quantification method. It was observed that the expression level of Alu RNA was not significantly different between GA and Control groups (median = 21.3 in both GA and Control groups, P = 0.251). In addition, the systemic level of Alu RNA was not associated with subject characteristics, such as GA lesion size and SNP profiles of complement factors associated with increased risk of AMD. In conclusion, the usability of systemic Alu RNA expression level as a biomarker of GA secondary to AMD could not be established in this study.

## Introduction

Age-related macular degeneration (AMD) is one of leading causes of blindness in elderly people in the United States [1]. In early and intermediate AMD, drusen, protein, and extra-cellular deposits between the retinal pigment epithelium (RPE) and Bruch’s membrane are observed [2]. Many patients with intermediate AMD progress to the advanced stage. In advanced AMD, geographic atrophy (GA) and/or choroidal neovascularization (CNV) are observed [2]. GA secondary to AMD, is characterized by an irreversible loss of macular retinal tissue and RPE cells, and is a cause of central visual function loss. No treatment is available to prevent or reverse visual function loss secondary to GA [3–5].

AMD is a multi-factorial and complex disease. Several genetic factors, including single-nucleotide polymorphisms (SNPs) and the affected genes, have been reported to be associated with AMD [6]. The intracellular accumulation of lipofuscin, including N-retinylidene-N-retinylethanolamine (A2E), in the RPE cells causes their death [7]. Drusen, which are extracellular deposits, are composed of cellular waste products, lipids, lipoproteins, and amyloid deposits. The components of these deposits trigger inflammation and are regulated by several cascades, including the complement pathway and NLRP3 inflammasome. This inflammation leads to GA [7–8]. Therefore, a number of therapies have been considered for these risk-factors in the past. Particularly, complement-based therapeutics directed against GA, such as those using an anti-CFH antibody and C3 inhibitors, have been under development globally [8]. In 2005, four research groups revealed that SNPs of *complement factor H* (*CFH*) are associated with an increased risk of developing AMD [9–12]. In addition, other SNPs in the *complement pathway*, such as in *complement component C3* (*C3*), *complement component C2* (*C2*)/*complement factor B* (*CFB*), and *complement factor I* (*CFI*), are also associated with an increased risk of AMD [6]. The relationships between these SNPs and GA have been analyzed in several clinical trials [13–15]. These SNPs are anticipated to serve as predictive markers for GA in clinical settings.

Alu RNAs are classified as short interspersed nuclear elements and are transcribed from *Alu* elements in retrotransposons [16]. These elements are the most abundant repetitive elements in the human genome and are approximately 300 base pairs long [16]. Recent studies revealed that GA patients showed a decreased level of DICER1, a micro RNA-processing enzyme, in their RPE. Insufficient DICER1 expression in RPE cells leads to the accumulation of Alu RNA in these cells, which results in their degeneration. Alu RNA might play a key role in the death of RPE cells and in the development of GA pathology [17–18]. Thus, Alu RNA and its related pathways could be promising targets for the treatment of GA and the detection of Alu RNA should be a useful marker for selection of patients for these treatments. As it is not realistic to obtain patients’ ocular samples, one practical way could be to detect ocular Alu RNA in the systemic blood. However, there are no reports of determination of systemic Alu RNA levels in patients with GA. Furthermore, there is difficulty in the measurement of Alu RNA in the blood because of contamination of genomic *Alu* sequence. Thus, in the present study, we investigated the relationship between systemic expression of Alu RNA and the pathology of GA associated with AMD using a novel proprietary method to quantify the expression levels of Alu RNA in the blood that enables us to avoid the contamination of genomic *Alu* sequence.

## Materials and methods

This study was a non-interventional, cross-sectional study, assessing the feasibility of using systemic Alu RNA expression level as a biomarker of GA in patients with AMD. The duration of this clinical study was up to 7 days. Blood samples were collected from eligible subjects on Day 1. Other assessments were made within 7 days prior to Day 1.

### Ethics

The study was conducted as per the guidelines of Declaration of Helsinki and was approved by the institutional review boards at Research Ethic Committee of Santen Pharmaceutical (Approval number: RINRI096) and Chesapeake IRB (Approval numbers: SSU00036567, SSU00037012, and SSU00037010). All the subjects were enrolled at three clinical sites in the United States upon obtaining the approval from Chesapeake IRB. Written informed consent was obtained from all the study participants. Also, the sample analysis and statistical analysis were performed at Santen Pharmaceutical Co. Ltd. after obtaining the approval from Research Ethic Committee of Santen Pharmaceutical.

### Subjects

The subjects (≥ 50 years old) were enrolled in GA and Control groups. The subjects with a medical history of exposure to factors that alter the expression of Alu RNA, such as those using glibenclamide, drug treatment for depression, or those who were diagnosed to have Alzheimer’s disease, were excluded from both the groups. The patients with GA secondary to AMD in either eye, and without a history of other retina diseases, were enrolled in GA group. The inclusion criterion of total GA lesion size was from 1 disc area (DA, 2.5 mm^2^) to 7 DA (17.5 mm^2^) in either eye. The subjects without AMD, more severe than level 1 (AREDS scale), a history of other retina diseases, and/or a history of other severe ophthalmic diseases were enrolled in Control group.

### Ophthalmic assessments

To confirm the eligibility and characteristics of subjects, they received ophthalmic assessments of both eyes (OU), which included best-corrected visual acuity (BCVA, Snellen visual acuity), slit-lamp biomicroscopy, indirect ophthalmoscopy, and fundus autofluorescence (FAF) imaging (for GA group only).

### FAF imaging and measurement of GA lesion size

FAF imaging was performed for the GA group only. The GA lesion area was defined as the area of decreased FAF signal detected by the fluorescein angiography feature of Confocal Scanning Laser Ophthalmoscopy (cSLO) system (Spectralis HRA, Heidelberg Engineering, Heidelberg, Germany or similar cSLO system). The size of the GA lesion was semi-automatically measured using Heidelberg Eye Explorer software or its equivalent. The eye with the larger GA area (total sum of areas, in case of multiple areas of GA) was chosen as the study eye.

### Genotyping of SNPs in the genes of the complement pathway

This analysis was performed only for the subjects in the GA group. Whole blood sample (8.5 mL) was collected from the subjects in the GA group in PAXgene Blood DNA tubes (Becton, Dickinson and Company, Franklin Lakes, NJ, USA) at Day 1. DNA was extracted from whole blood samples using PAXgene Blood DNA Kit (Qiagen, Hilden, Germany). Genotyping of SNPs in the genes of the complement pathway was performed by TaqMan SNP genotyping assays (Thermo Fisher Scientific Inc., Waltham, MS, USA). Six SNPs were confirmed in the complement pathway (Table 1).

**Table 1:** Single Nucleotide Polymorphisms (SNPs) in the genes of complement pathway associated with geographic atrophy secondary to age-related macular degeneration.

Gene	Reference SNP ID number (rs ID)
***CFI***	rs17440077
	rs4698775
***C3***	rs2230199
***C2*/*CFB***	rs429608
***CFH***	rs1329428
	rs1061170

### Measurement of systemic Alu RNA levels

In a previous study, it was indicated that systemic expression levels of Alu RNA have diurnal variations [19]. Therefore, we collected 10 mL of whole blood sample using Cell-Free RNA BCT (Streck, La Vista, NE, USA) at 10:00 AM (± 2 h). Cell-free plasma was extracted from the whole blood sample according to the instructions provided with the blood collection tube. Thereafter, the expression level of Alu RNA was measured by a novel quantitative RT-PCR (Reverse Transcription–Polymerase Chain Reaction) method (Santen Pharmaceutical Co., Ltd. Research and Development Center, Nara, Japan) using specially designed primers that precluded the risk of amplification of contaminating *Alu* genomic DNA. In brief, total RNA was extracted from 0.2 mL of plasma sample using miRNeasy Serum/Plasma Kit (Qiagen, Hilden, Germany) and miRNeasy Serum/Plasma Spike-In Control (Qiagen, Hilden, Germany). The extracted RNA samples were reverse transcribed to cDNA using miScript II RT kit (Qiagen, Hilden, Germany). The cDNA was used as a template for quantitative real-time PCR that was performed on ABI Prism 7500 Fast Sequence Detection System. Quantification of Alu RNA was performed using miScript SYBR Green PCR kit (Qiagen, Hilden, Germany) and Alu RNA specific forward primer (5'-CAA CAT RGT GAA ACC CCG TCT CT-3′). The synthesized RNA was used to normalize the data for the standard sample. Data set was normalized by the synthesized RNA, that had Alu RNA sequence, and miRNeasy Serum/Plasma Spike-In Control and then the copy number was calculated.

### Statistical analysis

SAS software (SAS Inc., Cary, NC, USA) was used for statistical analyses. All measurements were summarized by subject group (GA and Control groups), descriptively. The expression levels of Alu RNA were determined for each group using log transformation. The analysis methods are summarized in Table 2.

**Table 2:** Summary of statistical analysis methods used in the study.

Purposes	Methods
Comparison of expression levels of Alu RNA between GA and Control Groups	Unpaired *t*-test and analysis of covariance (ANCOVA)
Comparison of expression levels of Alu RNA between GA and Control Groups (Sub-group analysis)	Descriptive statistics and calculation of 95% confidence interval (CI)
Correlation between the expression levels of Alu RNA and BCVA	Pearson’s correlation analysis
Correlation between the expression levels of Alu RNA and GA lesion size	Pearson’s correlation analysis
Comparison of the expression levels of Alu RNA according to the presence of each SNP	Unpaired *t*-test

## Results

### Demographics and characteristics of the subjects

A total of 73 subjects were enrolled for the study; 33 subjects were enrolled in GA group and 40 were enrolled in Control group. The demographics and characteristics of the subjects are summarized in Table 3. The mean age (SD) was 80.1 (8.1) years in GA group and 59.1 (6.2) years in Control group, it being statistically higher in the former (P < 0.05). The percentage of females was 66.7% in GA group and 77.5% in Control group. Most of the subjects were of white race in both the groups (GA group: 93.9%, Control group: 85.0%). The percentage of former or current smokers in GA group (48.5%) was higher than that in Control group (27.5%). The mean BCVA of the study eye in GA group (SD) was 0.282 (0.249).

**Table 3:** Demographics and characteristics of the subjects.

		GA Group (N = 33)	Control Group (N = 40)
**Age, years, mean (SD)**		80.8 (8.1)	59.1 (6.2)
**Sex, N (%)**	Male	11 (33.3)	9 (22.5)
	Female	22 (66.7)	31 (77.5)
**Race, N (%)**	White	31 (93.9)	34 (85.0)
	Asian	1 (3.0)	5 (12.5)
	Other	-	1 (2.5)
	Not reported	1 (3.0)	-
**Smoking status, N (%)**	Never smoked	17 (51.5)	29 (72.5)
	Former smoker	15 (45.5)	8 (20.0)
	Current smoker	1 (3.0)	3 (7.5)
**Family history of AMD, N (%)**	Yes	6 (18.2)	9 (22.5)
	No	11 (33.3)	30 (75.0)
	Unknown	16 (48.5)	1 (2.5)
**BCVA, mean (SD)**	Right eye	0.308 (0.274)	0.855 (0.279)
	Left eye	0.361 (0.266)	0.852 (0.292)
	Study eye	0.282 (0.249)	-
**GA lesion size (mm**^**2**^**)**	Mean (SD)	9.685 (4.983)	-
	Median	8.470	-
	Min, Max	3.14, 21.98	-

### Expression level of Alu RNA and between-the-groups comparison

The mean expression level (log transformation) (SD) of Alu RNA was 21.4 (0.6) in GA group and 21.2 (0.8) in Control group whereas the median level was 21.3 in both the groups. There was no significant between-the-groups difference (P = 0.251) (Table 4).

**Table 4:** Expression levels (log transformation) of Alu RNA.

	GA Group (N = 33)	Control Group (N = 40)
**Mean (SD)**	21.4 (0.6)	21.2 (0.8)
**Median**	21.3	21.3
**Min, Max**	20, 23	19, 23
**P-value**		0.251

There were between-the-groups differences in subjects’ age and sex; adjusted between-the-groups comparison was conducted using ANCOVA. However, the results of ANCOVA showed that there was no between-the-groups difference in the expression levels (log transformation) of Alu RNA, adjusted for age or sex (Table 5).

**Table 5:** Comparison of the expression levels (log transformation) of Alu RNA in the groups as assessed using ANCOVA.

Adjusted by		GA Group (N = 33)	Control Group (N = 40)
Age	Least Square Mean (SE)	21.32 (0.20)	21.20 (0.17)
	P-Value		0.703
Sex	Least Square Mean (SE)	21.37 (0.13)	21.16 (0.12)
	P-Value		0.252

Furthermore, there was no between-the-groups difference in the expression levels (log transformation) of Alu RNA in any of the sub groups (Table 6).

**Table 6:** Sub group analysis of the expression levels (log transformation) of Alu RNA.

Sub group		Categories	GA Group	Control Group	GA Group—Control Group
Age	Mean (SD)	<65	-	21.20 (0.87)	
	N		N = 0	N = 33	
	Mean difference (95% CI)				-
	Mean (SD)	65< =	21.37 (0.63)	21.02 (0.68)	
	N		N = 33	N = 7	
	Mean difference (95% CI)				0.35 (-0.18–0.89)
Sex	Mean (SD)	Male	21.28 (0.63)	21.24 (1.25)	
	N		N = 11	N = 9	
	Mean difference (95% CI)				0.04 (-0.87–0.95)
	Mean (SD)	Female	21.41 (0.64)	21.14 (0.70)	
	N		N = 22	N = 31	
	Mean difference (95% CI)				0.27 (-0.11–0.65)
Race	Mean (SD)	White	21.38 (0.64)	21.24 (0.70)	
	N		N = 31	N = 34	
	Mean difference (95% CI)				0.14 (-0.19–0.48)
	Mean (SD)	Asian	21.4 (-)	20.43 (1.30)	
	N		N = 1	N = 5	
	Mean difference (95% CI)				0.96 (-3.00–4.93)
	Mean (SD)	Other	- (0)	22.3 (-)	
	N		N = 0	N = 1	
	Mean difference (95% CI)				-
	Mean (SD)	Not reported	20.9 (-)	-	
	N		N = 1	N = 0	
	Mean difference (95% CI)				-
Smoking status	Mean (SD)	Never smoked	21.41 (0.67)	21.12 (0.80)	
	N		N = 17	N = 29	
	Mean difference (95% CI)				0.28 (-0.18–0.75)
	Mean (SD)	Former smoker	21.34 (0.61)	21.36 (0.88)	
	N		N = 15	N = 8	
	Mean difference (95% CI)				-0.02 (-0.67–0.63)
	Mean (SD)	Current smoker	21.1(-)	21.03 (1.33)	
	N		N = 1	N = 3	
	Mean difference (95% CI)				0.10 (-6.50–6.70)
Family history of AMD	Mean (SD)	Yes	21.32 (0.65)	21.16 (0.63)	
	N		N = 6	N = 9	
	Mean difference (95% CI)				0.16 (-0.57–0.88)
	Mean (SD)	No	21.31 (0.87)	21.14 (0.90)	
	N		N = 11	N = 30	
	Mean difference (95% CI)				0.17 (-0.46–0.81)
	Mean (SD)	Unknown	21.42 (0.44)	22.0 (-)	
	N		N = 16	N = 1	
	Mean difference (95% CI)				-0.53 (-1.50–0.45)

### Relationship between the expression levels of Alu RNA and BCVA

In GA group, BCVA in the right eye was very weakly correlated with the expression level of Alu RNA (correlation coefficient = -0.307). In the left eye in GA Group and in each of the eyes in Control Group, BCVA was not associated with the expression level of Alu RNA (Table 7).

**Table 7:** Correlation between the expression levels (log transformation) of Alu RNA and BCVA.

	Correlation coefficient		
Eye	GA Group	Control Group	All Subjects
Right	-0.307	-0.020	-0.185
Left	-0.052	-0.053	-0.129

### Relationship between the expression levels of Alu RNA and the size of GA lesion

In GA group, the size of GA lesion in the study eye was measured using the FAF images. The mean GA lesion size (SD) was 9.69 (4.98). The correlation between the expression level of Alu RNA and the total GA lesion size was also analyzed; we did not find any correlation between the two (correlation coefficient = 0.126).

### Relationship between the expression of Alu RNA and SNPs in the risk alleles associated with the complement pathway

The genotyping of genes associated with the complement pathway was performed in GA group. The heterozygotes and homozygotes for the risk alleles were defined as “Risk-allele Yes” because of the small sample size in this study. All the subjects had the *C2*/*CFB* risk-allele, except for one subject for whom it could not be determined. All the subjects had the *C3* risk-allele. The expression levels of Alu RNA in the presence or absence of the risk-allele were compared for *CFI* and *CFH*. No significant differences in the expression levels of Alu RNA were observed among the subgroups (Table 8).

**Table 8:** Expression levels of Alu RNA in the presence and absence of the risk-alleles.

			Risk-allele	
Gene	rs ID	Expression level of Alu RNA	Yes	No
***CFI***	rs17440077	N (%)	14 (42.4)	19 (57.6)
		Mean (SD)	21.3 (0.6)	21.4 (0.7)
		Median	21.4	21.2
		Min, Max	20, 23	20, 23
		P-value	0.817	-
***CFI***	rs4698775	N (%)	15 (45.5)	18 (54.5)
		Mean (SD)	21.4 (0.6)	21.4 (0.7)
		Median	21.4	21.2
		Min, Max	20, 23	20, 23
		P-value	0.973	-
***C3***	rs2230199	N (%)	33 (100)	0 (0)
		Mean (SD)	21.4 (0.6)	-
		Median	21.3	-
		Min, Max	20, 23	-
		P-value	-	-
***C2/CFB***	rs429608	N (%)	32* (97.0)	0 (0)
		Mean (SD)	21.4 (0.6)	-
		Median	21.3	-
		Min, Max	20, 23	-
		P-value	-	-
***CFH***	rs1329428	N (%)	32 (97.0)	1 (3.0)
		Mean (SD)	21.4 (0.6)	20.9 (-)
		Median	21.3	20.9
		Min, Max	20, 23	21, 21
		P-value	0.507	-
***CFH***	rs1061170	N (%)	26 (78.8)	7 (21.2)
		Mean (SD)	21.3 (0.5)	21.7 (0.9)
		Median	21.3	21.5
		Min, Max	20, 22	21, 23
		P-value	0.082	-

* One subject could not be evaluated for the *C2/CFB* risk-allele.

## Discussion and conclusion

This is the first study to analyze the relationship between the systemic expression levels of Alu RNA and GA secondary to AMD. Previous studies indicated that the expression of Alu RNA in the ocular tissue is associated with degeneration of RPE and development of AMD [17–18]. However, no difference in the systemic expression of Alu RNA was observed between the GA and Control groups. Furthermore, based on the results of analysis of covariance and sub-group analysis, adjusting between-the-groups differences in subjects’ background, we did not find any difference in the systemic expression of Alu RNA between GA and Control Groups. These results indicate that detection of the systemic expression of Alu RNA is not suitable as a selection marker for GA secondary to AMD.

The relationships between the systemic expression levels of Alu RNA and ocular conditions were also investigated. In GA group, BCVA in the right eye was very weakly correlated with the systemic expression levels of Alu RNA; however, this was not the case for the left eye in this group. Overall, the expression level of Alu RNA was not associated with BCVA in both the eyes. If the systemic Alu RNA levels were associated with BCVA in one eye, they should also be correlated with BCVA in the other eye. Therefore, the observed weak correlation in only the right eye cannot be considered to have any meaning.

SNPs in the risk alleles associated with the complement pathway are associated with an increased risk of developing AMD [6,9–12]. The genotyping of such SNPs associated with GA secondary to AMD was performed in several clinical trials [13–15]. However, no relationship between these SNPs and systemic expression levels of Alu RNA was observed. Therefore, we compared the systemic expression levels of Alu RNA between the presence and absence of risk allele for *CFI* and *CFH* but did not find any difference in the expression levels. These results indicate that risk alleles for *CFI* and *CFH* are not associated with the systemic expression of Alu RNA. The Alu RNA pathway may independently associate with the degeneration of RPE through the alternative complement pathway, especially *CFI* and *CFH*. The relationship between the expression level of Alu RNA and *C2/CFB* and *C3* was not analyzed because all eligible subjects had these risk alleles.

In conclusion, the usability of systemic Alu RNA expression level as a biomarker of GA secondary to AMD could not be established in this study. For further investigation of the utility of Alu RNA as a biomarker, the relationship between the expression levels of Alu RNA in ocular tissues and GA lesion size and/or rate of progression should be investigated. Chorioretinal tissues are ideal for the investigation; however non-/less-invasive collection of such a sample is difficult and not suitable for biomarker studies, and is therefore not possible in clinical settings. Although their feasibility is unknown, methods for measuring the expression levels of Alu RNA or related cytokines, such as IL-18, in the anterior aqueous humor and tear fluid, or novel bioimaging technologies for detecting biomarkers in chorioretinal tissues are warranted.
